# Engineered *Bacillus subtilis* WB600/ZD prevents *Salmonella* Infantis-induced intestinal inflammation and alters the colon microbiota in a mouse model

**DOI:** 10.1186/s13567-024-01438-z

**Published:** 2025-02-08

**Authors:** Wei Li, Xue Wang, Keyuan Chen, Yaohong Zhu, Guiyan Yang, Yipeng Jin, Jiufeng Wang

**Affiliations:** 1 College of Veterinary Medicine, China Agricultural University, Beijing, 100193 China; 2 Sanya Institute of China Agricultural University, Sanya, 572025 Hainan China; 3 College of Veterinary Medicine, Inner Mongolia Agricultural University, Hohhot, 010000 China

**Keywords:** *Zophobas atratus* defensin, engineered bacteria, *B. subtilis* WB600/ZD, *S.* Infantis infection, small intestinal inflammation

## Abstract

Antimicrobial peptides (AMPs) are instrumental in maintaining intestinal homeostasis and have emerged as potential therapeutic candidates for ameliorating intestinal bacterial infections. However, the intrinsic instability associated with the in vivo delivery of AMPs constitutes a substantial impediment to their therapeutic efficacy in treating infections. In this study, we genetically modified *Bacillus subtilis* (*B. subtilis*) WB600 to express *Zophobas atratus* defensin (ZD), an antimicrobial peptide with broad-spectrum activity isolated from *Zophobas atratus*, for oral administration. This engineered strain effectively protects against *Salmonella* Infantis (*S.* Infantis) infection in mice. Pretreatment with WB600/ZD prevented NF-κB pathway activation induced by *S.* Infantis infection and increased expression of antioxidant and tight junction proteins, thus alleviating the severity of intestinal inflammation in both the jejunum and ileum (*P* < 0.01). Moreover, WB600/ZD pretreatment facilitated the growth of beneficial bacteria such as *Lachnospiraceae*, *Butyricicoccus*, *Eubacterium_xylanophilum*, and *Clostridia_UCG-014* while decreasing the abundance of pathogenic bacteria such as *Escherichia-Shigella* and *Salmonella* (*P* < 0.05). In conclusion, this study underscores the protective effects of WB600/ZD on *S.* Infantis-induced intestinal inflammation, suggesting that oral delivery of *B. subtilis* WB600/ZD may be a promising prophylactic strategy for combating bacterial infections in the intestine.

**Supplementary Information:**

The online version contains supplementary material available at 10.1186/s13567-024-01438-z.

## Introduction

Antimicrobial peptides (AMPs), known as host defense peptides, are endogenously synthesized by various cells and tissues across nearly all complex organisms to sustain host‒microbe equilibrium [1, 2]. In addition to their antimicrobial properties, AMPs also possess immunomodulatory functions [3]. ZD, an antimicrobial peptide identified in previous studies conducted by our research team within the hemolymph of *Zophobas atratus*, has exhibited significant bacteriostatic activity against various pathogenic bacteria, including *Escherichia coli*, *Klebsiella pneumoniae*, and *Staphylococcus aureus*, among others. Consistent with the bactericidal mechanisms of most other cationic antimicrobial peptides, this peptide interacts electrostatically with anionic bacterial membranes, leading to disruption of the membrane potential, alteration of membrane permeability, and metabolite leakage, ultimately leading to bacterial cell death [4]. The therapeutic and prophylactic efficacy of ZD against bacterial infections has been validated in mouse models of *E. coli*-induced mastitis and scald [5, 6]. Indeed, the synthesis of defensins can be challenging because of their low yield and high cost. Additionally, their structure and function can be compromised by environmental factors, such as gastric acid, which leads to the degradation of oral defensins before they reach the gut [7, 8]. *B. subtilis* is commonly used in food fermentation as a safe probiotic for human consumption and can survive in the gastrointestinal tract [9]. This bacterium has been utilized as a carrier to express AMPs, such as cathelicidin-related antimicrobial peptide *KR32* or porcine β-defensin-2 (*pBD-2*), both of which effectively alleviate the intestinal inflammation induced by pathogenic bacteria [10, 11]. Thus, constructing ZD-producing *B. subtilis* may overcome the difficulties associated with AMP extraction and intestinal delivery, improving the potential effects of ZD on the protection of the intestine from enteric infection.

*Salmonella* Infantis (*S.* Infantis), a zoonotic pathogen, poses a serious risk to both humans [12] and affects various animals, particularly livestock such as pigs, poultry, and cattle [13, 14]. *S*. Infantis infection causes mild to severe gastrointestinal issues, including diarrhea, decreased growth rates, and even systemic infection, leading to reduced productivity and a greater risk of secondary infections [15]. In addition, an increasing number of *S*. Infantis isolates are multidrug resistant strains. Therefore, treating *S*. Infantis infectious diseases, especially severe or prolonged infections in humans and animals, is challenging [16, 17].

Probiotics, as promising alternatives to antibiotics, can mitigate the effects of *S*. Infantis through multiple mechanisms [18–21]. They help restore the gut microbiota balance, enhance the host immune system [14], produce antimicrobial substances, and improve intestinal barrier integrity, thereby reducing pathogen colonization and infection risk [22]. Integrating probiotics into animal husbandry practices can reduce the need for antibiotics and improve animal welfare and productivity.

Here, we constructed the engineered probiotic bacteria WB600/ZD as a vector to express and secrete ZD and demonstrated the beneficial effect of WB600/ZD in a mouse model of small intestinal inflammation induced by *S.* Infantis.

## Materials and methods

### Strains, plasmids, and bacterial cultures

*S.* Infantis CAU1508 was isolated from the intestinal contents of diarrheal piglets [20]. LB and XLT4 media were purchased from HOPEBIO (Qingdao, China). *B. subtilis* WB600, *B. subtilis* BS168, and the PMA5 plasmid were obtained from Wuhan Mioling Biotechnology Co., Ltd. (Wuhan, China). Trans5α chemically competent cells were obtained from TransGen (Beijing, China).

### Chemicals

The following primary antibodies were used for western blot, immunofluorescence and immunohistochemical staining: HIS-tag, β-Actin, Claudin-1, Occludin, ZO-1, HO-1, Nrf2, IκB, p-IκB, P65 and p-P65 were purchased from Proteintech (Wuhan, China). Alexa Fluor 488 polyclonal goat anti-rabbit IgG (H + L) was purchased from Beyotime Biotechnology (Shanghai, China). A DAB Horseradish Peroxidase Color Development Kit and enhanced enzyme-labelled goat anti-rabbit IgG polymer were purchased from Beijing Zhongsui Jinqiao Biotechnology Co., Ltd. (Beijing China). The following primary primers were used for PCR and RT‒qPCR: *SPbpr*, *SPdacB*, *SPeapD*, *SPlytE*, *SPwapA*, *GAPDH*, *IL-6*, *IL-1β*, *TNF-α*, *MUC2*, *TFF3*, and *GAL3ST2* were synthesized by Sangon Biotech Co., Ltd. (Shanghai, China). The kits used in this study included malondialdehyde (MDA), catalase (CAT), myeloperoxidase (MPO), glutathione peroxidase (GSH-PX), superoxide dismutase (SOD), and total antioxidant capacity (T-AOC) ELISA kits; RIPA lysis buffer and PMSF were purchased from Solarbio (Beijing, China). A BCA protein assay kit was purchased from Beyotime Biotechnology (Shanghai, China). QuickCut *Bam*H I and TRIzol reagents were purchased from TaKaRa (Kyoto, Japan). The reverse transcription reagent kit and SYBR qPCR Master Mix were purchased from TransGen (Beijing, China).

### Construction and identification of WB600/ZD expressing ZD

WB600 was incubated overnight at 37 °C in SPI medium. The following day, 100 µL of the culture was inoculated into 5 mL of fresh SPI medium. Upon reaching an OD_600_ of 0.8, 200 µL was promptly transferred into 2 mL of SPII medium. The culture was then shaken at 100 rpm at 37 °C for 1.5 h. Subsequently, 20 µL of a 100 × EGTA solution was added, followed by incubation at 37 °C with shaking at 100 rpm for 10 min. The contents were dispensed into 1.5 mL centrifuge tubes at 500 µL per tube. PMA5 and PMA5-SPn-ZD plasmids at a final concentration of 1 µg/mL were added to the tubes. After gentle mixing, the tubes were incubated at 37 °C with shaking at 100 rpm for 30 min. They were then transferred to a shaking bed at 180 rpm and incubated at 37 °C for 1.5 h. The bacteria were collected by centrifugation at 4000 rpm, and part of the supernatant was discarded, leaving 100 µL for resuspension of the bacteria. This suspension was then applied to the corresponding selective medium and incubated overnight at 37 °C. Single colonies were selected for PCR verification, and PCR-positive bacterial mixtures were chosen for culture expansion and storage. The primers used for plasmid construction can be found in Additional file 1. The constructed expression strain was inoculated in 2 × SR medium and incubated at 37 °C and 180 rpm for 24 h. The supernatant was collected for western blot analysis of the target protein.

### Animals

Male C57BL/6 J mice aged 6–8 weeks were obtained from Sipeifu Biotechnology Co., Ltd. (Beijing, China) and housed at the Animal Housing Unit of China Agriculture University in Beijing, China. The mice were kept under controlled temperature conditions (23–25 °C) with a 12-h light‒dark cycle. All experimental procedures involving animals were approved by the Animal Ethics Committee of China Agriculture University under protocol number AW10304202-2-1.

### Experimental design

Following a three-day acclimatization period, male C57BL/6 J mice were randomly allocated into the following experimental groups (*n* = 8 per group): the control group (CON), *S.* Infantis infection group (SI), WB600 pretreatment group (WB600 + SI), and WB600/ZD pretreatment group (WB600/ZD + SI). The CON group received 200 µL of PBS via gavage from day 1 to day 17. The SI group was administered 200 µL of PBS via gavage from day 1 to day 14, followed by the administration of 200 µL (1 × 10^6^ CFU) of *S.* Infantis by gavage from day 15 to day 17. From days 1 to 14, the WB600 + SI and WB600/ZD + SI groups were orally administered 200 µL (1 × 10^6^ CFU) of WB600 and WB600/ZD bacterial solutions, respectively. They were then given 200 µL (1 × 10^6^ CFU) of *S*. Infantis by gavage from day 15 to day 17. On day 21, the mice were euthanized, and tissue samples were collected for subsequent analyses.

### Clinical indicator detection

Body weight and temperature were monitored at the same time every day during the trial. The abdominal temperature of the mice was measured via an electronic thermometer, and the mice were depilated at the test site before the test. Approximately 0.5 g of feces was collected and dried at 60 °C for 24 h, after which the weight of the feces was tested. The ratio of the dried feces to the weight before drying was calculated. The criteria for scoring faeces were as follows: 0–1, normal; 2–3, soft or shapeless; and 4–5, loose or watery stools. The colon, liver and spleen were collected after the mice were euthanized on day 21. The tissue weights and colon lengths were recorded.

### Fecal *S*. Infantis load

On days 1–3 post infection, faecal samples weighing approximately 0.5 g were collected in 5 mL centrifuge tubes. To each tube, 4.5 mL of sterile phosphate-buffered saline (PBS) was added, and the mixture was crushed and centrifuged at 450 × *g* for 1 min. The supernatant was carefully transferred to a new 1.5 mL sterile Eppendorf (EP) tube and centrifuged at 3600 × *g* for 15 min. The resulting supernatant was discarded, and the pellet was resuspended in 1 mL of sterile PBS, thoroughly mixed, and then subjected to three tenfold serial dilutions, with three replicates for each dilution. Subsequently, 100 μL of the diluted supernatant was evenly spread onto XLT4 agar plates. The plates were then incubated at 37 °C for 36–48 h until distinct black single colonies were fully developed, at which point they were enumerated.

### GI transit

On the third day after *S*. Infantis infection, three mice were randomly chosen from each experimental group. After a 24-h period of fasting without access to food or water, each mouse received a 200 μL suspension of charcoal powder. This suspension was prepared by dissolving 10 g of activated charcoal in 100 mL of PBS containing 2 g of carboxymethylcellulose sodium (CMC-Na). Following the administration of charcoal, the mice underwent an additional fasting and water deprivation period of 30 min. After the designated fasting period, the mice were euthanized, and their abdominal cavities were carefully opened. The mesentery was separated, and the small intestine was extracted and measured. The distance from the pylorus to the ileum was recorded as L1. The length of advancement of the charcoal powder along the small intestine was noted as L2. The small intestinal transit rate was calculated as a percentage using the following formula: Small Intestinal Transit Rate (%) = (L2/L1) × 100%.

### Real-time PCR

Total RNA was extracted from the jejunum and ileum tissues of the mice using TRIzol reagent in accordance with the recommended protocol provided by the manufacturer. Subsequently, complementary DNA (cDNA) was synthesized from the extracted RNA samples using a reverse transcription reagent kit. The cDNA samples were then subjected to amplification using the Applied Biosystems 7500 Fast Real-Time PCR System in conjunction with SYBR qPCR Master Mix and specific primers targeting the genes of interest. The quantification of gene expression levels was performed utilizing the 2^−ΔΔct^ method, which involves normalization to the housekeeping gene *GAPDH*. The sequences of primers used for the amplification of the mouse genes were sourced from the National Center for Biotechnology Information (NCBI) and were custom synthesized by Sangon Biotech Co., Ltd. (Shanghai, China). A detailed list of the sequences of primers used for the RT‒qPCR analysis can be found in Additional file 2.

### Western blotting

The ileum tissues were homogenized in ice-cold RIPA lysis buffer containing PMSF. The supernatant was subsequently obtained by centrifugation (12 000 × *g*, 4 °C, 15 min), and the protein concentration was quantified using a BCA protein assay kit. All the samples were loaded with equal amounts of protein, separated by electrophoresis on SDS polyacrylamide gels and then transferred onto PVDF membranes. The membranes were blocked with skim milk for 1.5 h, further incubated with primary antibody overnight at 4 °C, and then incubated with a secondary peroxidase-labelled antibody for 1 h. Grayscale analysis of protein expression was performed using a gel imaging analyser (Tanon, Shanghai, China) and a gel imaging system (Tanon).

### Measurement of the activities of antioxidant enzymes

On day 21 of the experiment, blood samples were collected from the mice and centrifuged at 4000 × *g* for 10 min to separate the serum. The collected serum was then utilized directly for the assessment of antioxidant enzyme activities. The assays for MDA, CAT, MPO, GSH-PX, SOD and T-AOC were carried out according to the instructions provided by the manufacturers.

### Immunohistochemical staining

The colon tissues were fixed in 4% paraformaldehyde, embedded in paraffin, and sectioned into slices with a thickness of 3 μm. After dewaxing and rehydrating, the tissue sections were subjected to antigen retrieval by incubation in citrate buffer (pH 6) at 100 °C for 20 min using a steamer. Following blocking with 5% BSA for 30 min at room temperature, the samples were stained with rabbit polyclonal antibodies against MUC2 and incubated overnight at 4 °C. A histochemical secondary antibody kit was subsequently used for 1 h at room temperature. Finally, coverslips were applied after adding drops of neutral resin.

### Immunofluorescence staining

ZO-1 was detected in the ileum, and sections were prepared for immunohistochemistry. The samples were stained with rabbit polyclonal antibodies against ZO-1 and incubated overnight at 4 °C. The secondary antibody was detected using incubation with Alexa Fluor 488 polyclonal goat anti-rabbit IgG (H + L) for 1 h at room temperature. The samples were incubated with DAPI at room temperature for 10 min to stain the nuclei. The cover slips were mounted with anti-fade mounting medium.

### Histological analysis

Mouse tissues were fixed in 4% paraformaldehyde for 24 h. The tissues were then dehydrated and embedded in paraffin according to standard procedures to prepare sections. Sections were stained with hematoxylin and eosin to analyse the histological properties of the tissues.

### Pathological scoring criteria

Tissues from mice not infected with *S.* Infantis were used as negative controls. Pathological changes were observed under a microscope and scored on the basis of the severity of lesions from 0 to 4. The scoring criteria were as follows: 0 = no visible damage; 1 = minor lesions with mild edema and epithelial cell detachment; 2 = mild lesions with mild inflammation and some inflammatory cell infiltration around blood vessels; 3 = moderate lesions with more inflammatory cell infiltration and significant structural damage around blood vessels; and 4 = severe lesions with intense inflammatory cell infiltration and severe structural damage around blood vessels.

### 16S rRNA gene sequencing and KEGG pathway analysis of the predicted gut microbiota

Colonic contents from the mice were frozen in liquid nitrogen for preservation. Microbial DNA was isolated from the colonic contents, and the hypervariable region of 16S rRNA was amplified. The purified amplicon sequences were analysed using Illumina MiSeq (PE300) sequencing. The KEGG pathways of the gut microbiota were predicted via PICRUSt2, which stands for phylogenetic investigation of communities by reconstruction of unobserved states. Data analysis was performed by Majorbio Biopharm Biotechnology for further investigation and interpretation.

### Statistical analysis

Statistical analysis and figure generation were conducted using GraphPad Prism 8. The data are presented as the means ± standard deviation (SD). To assess differences among three or more groups, one-way or two-way analysis of variance (ANOVA) was performed, followed by Dunnett’s multiple comparisons test. A *P* value of less than 0.05 was considered statistically significant.

## Results

### Construction of WB600/ZD and detection of ZD expression

**Figure 1:**
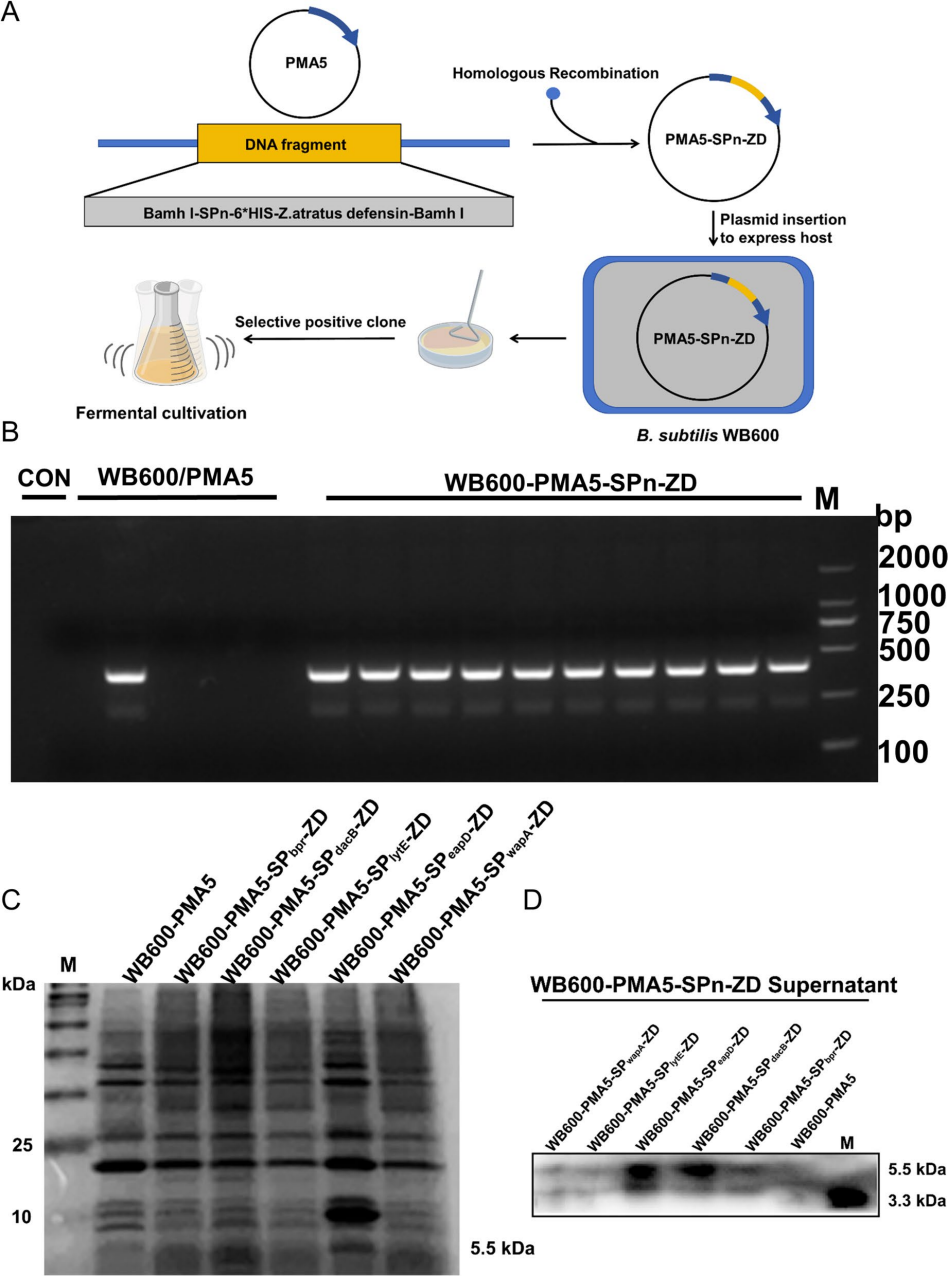
**WB600/ZD secretes ZD**. **A** Schematic representation of engineered WB600/ZD. **B** Positive transformants detected by polymerase chain reaction (PCR). **C**, **D** Coomassie Brilliant Blue staining and western blot analysis revealed the protein expression of ZD in the culture supernatant of WB600/ZD.

The recombinant plasmid and strain were initially engineered as depicted in Figure 1A. The signal peptide genes were PCR-amplified from BS168 (Additional file 3) and then fused with the ZD gene to create the complete SPn-ZD gene (Figure 1B). This recombinant plasmid was subsequently introduced into the WB600 system (Additional file 3). In the culture supernatant of the recombinant strain, a distinct band representing secreted ZD (5.5 kDa) was prominently detected through Coomassie Brilliant Blue staining and western blot analysis (Figures 1C and D). Finally, the bacteriostatic effect of the fermentation broth supernatant of the strain against pathogenic bacteria was investigated. The results revealed that WB600/ZD but not WB600 fermentation broth inhibited *S*. Infantis, *E. coli* ATCC25922, CAU201919, *Staphylococcus aureus* ATCC33591, ATCC29213, ATCC25923, and *Klebsiella pneumoniae* growth (Additional file 3). These results demonstrate the successful construction of the ZD expression system (WB600/ZD) and underscore the efficient guiding role of the *eapD* signal peptide in directing the secretion of the target protein. This achievement sets the stage for further investigations and applications in subsequent studies.

### Oral administration of WB600/ZD prevents *S*. Infantis-induced clinical symptoms in mice

**Figure 2:**
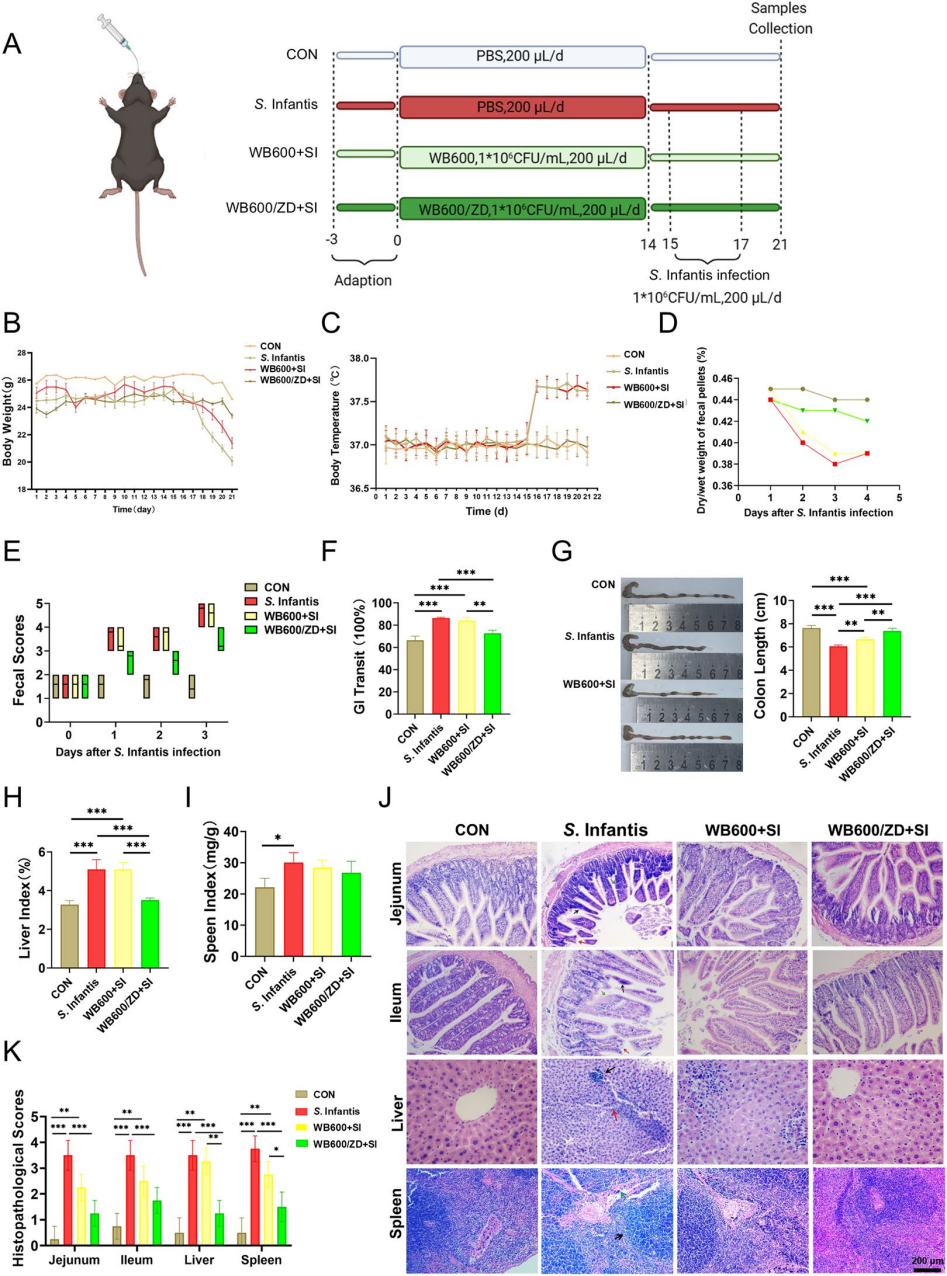
**Oral administration of WB600/ZD prevented** ***S.* Infantis-induced clinical symptoms**. **A** Animal experimental design. **B**, **C** Body weight and temperature changes were monitored over time. **D**, **E** The fecal wet/dry weight ratio and fecal scores were tested on three consecutive days after *S.* Infantis infection. **F** Gastrointestinal (GI) transit changes were assessed on day 21. **G–I** The colon length, spleen index, and liver index were measured at 4 days after *S*. Infantis infection. **J** Oral administration of WB600/ZD prevented histopathological damage caused by *S.* Infantis infection. In the jejunum and ileum, the black arrow indicates inflammatory cell infiltration of the intestinal villi, the red arrow indicates rupture of the intestinal villi, the green arrow indicates swelling of the intestinal villi, and the blue arrow indicates separation of the mucosal and muscular layers. In the liver, the black arrow indicates inflammatory cell infiltration, and the red arrow indicates disorganized liver cords. In the spleen, the black arrow indicates increased white marrow, and the green arrow indicates that the trabecular structure of the spleen is unclear (original magnification, 200 ×). All the data are presented as the means ± SD from three independent experiments. *P < 0.05, **P < 0.01, ***P < 0.001.

We subsequently examined the impact of WB600/ZD pretreatment on the development of intestinal inflammation. The experimental design involving mice is illustrated in Figure 2A. Mice in the SI group, without prior WB600/ZD pretreatment, presented significant increases in weight loss and increased body temperature. Notably, WB600/ZD, but not WB600, effectively prevented weight loss and increased body temperature in the mice (Figures 2B and C). WB600/ZD but not WB600 effectively prevented the decreases in fecal wet/dry weight ratios, increases in fecal scores, and changes in intestinal transit rates induced by *S.* Infantis infection (Figures 2D–F). Our study demonstrated that pretreatment with WB600/ZD effectively prevented the colon length shortening induced by S. Infantis infection compared with pretreatment with WB600 alone (Figure 2G). Furthermore, our findings indicated that *S.* Infantis infection leads to invasion of the liver and spleen in mice, resulting in elevated liver and spleen indices, a process that was effectively prevented by pretreatment with WB600/ZD (Figure 2H and I). Ultimately, pretreatment with WB600/ZD was found to reduce the fecal, liver, and spleen loads of *S.* Infantis (Additional file 4). Histological examination of the SI group confirmed severe damage caused by *S.* Infantis infection, including inflammatory cell infiltration (black arrow), rupture (red arrow), villus swelling (green arrow), and separation of the mucosal and muscular layers (blue arrow). Hepatic lesions were characterized by inflammatory cell infiltration (black arrow) and hepatocyte cord disorders (red arrow), whereas splenic lesions presented increased white marrow (black arrow) and unclear trabecular structure (green arrow). Pretreatment with WB600/ZD significantly reduced intestinal morphological changes and inflammatory infiltration and improved liver and spleen pathological changes induced by *S.* Infantis infection. Notably, WB600/ZD pretreatment led to reduced inflammatory cell infiltration in the liver, structural disorders in hepatocyte cords, and increased splenic leukomalacia caused by *S.* Infantis infection (Figure 2J).

### Oral administration of WB600/ZD prevents *S*. Infantis-induced intestinal barrier dysfunction

**Figure 3:**
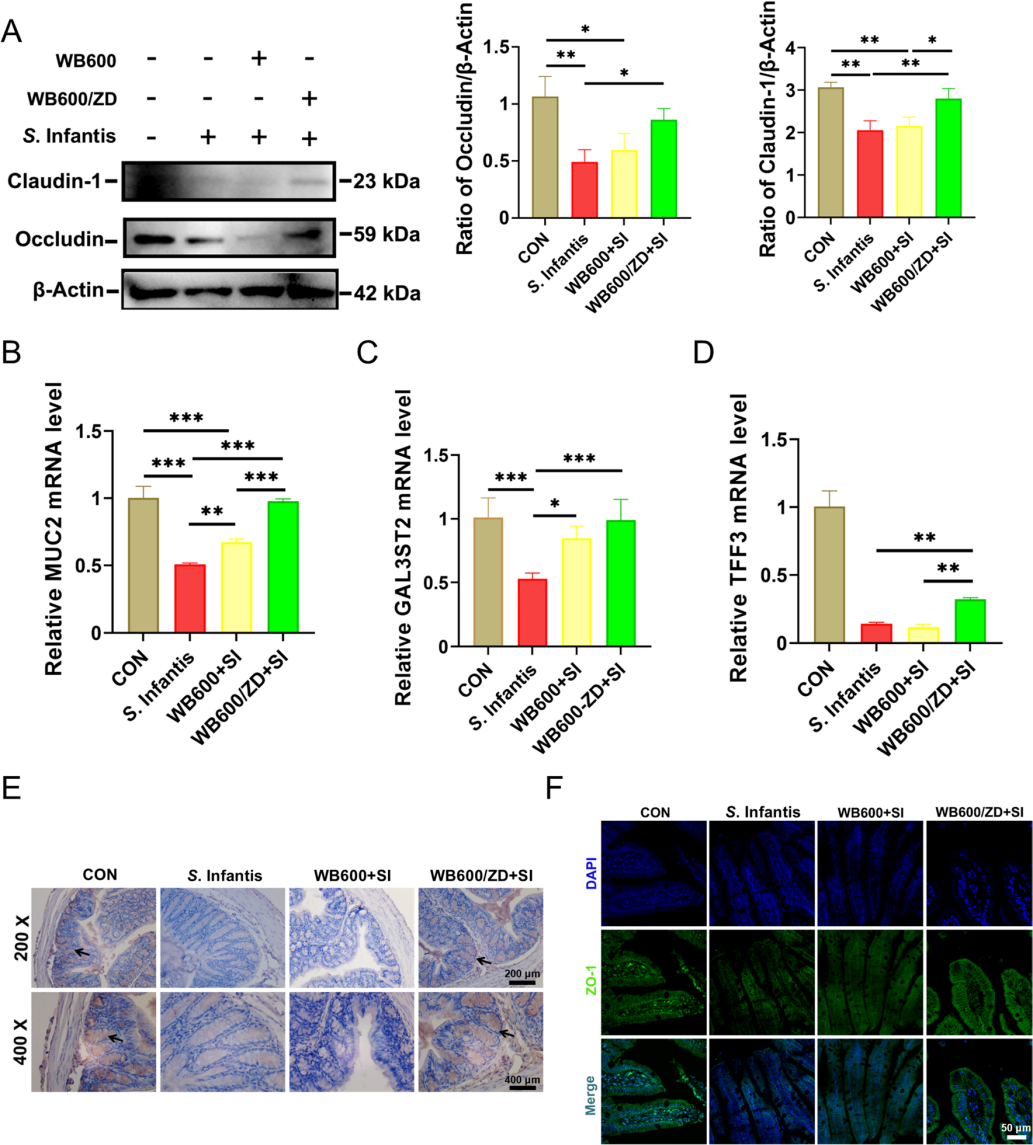
**Prefeeding with WB600/ZD prevented** ***S.* Infantis-induced intestinal barrier dysfunction**. **A** Western blot analysis of claudin-1 and occludin expression in the ileum. **B**–**D** Relative mRNA expression of *MUC2* and mucin-related *TFF3* and *GAL3ST2* in the ileum. **E** Immunohistochemical detection of MUC2 expression in the colon; black arrows indicate MUC2 distribution. **F** Immunofluorescence detection of ZO-1 expression in the ileum. All the data are presented as the means ± SD from three independent experiments. *P < 0.05, **P < 0.01, ***P < 0.001.

In the ileum, western blot analysis revealed that small intestinal claudin-1 and occludin in the SI group were severely downregulated compared with those in the CON group (Figure 3A). Intriguingly, pretreatment with WB600/ZD significantly increased the expression of TJPs in the ileum, whereas WB600 was less effective. The results revealed that in both the jejunum (Additional file 5) and ileum, pretreatment with WB600/ZD significantly preserved the expression of *MUC2*, *TFF3*, and *GAL3ST2* during *S*. Infantis infection, while WB600 pretreatment also had a certain alleviating effect, but it was far inferior to that of WB600/ZD (Figures 3B–D). Consistently, the immunohistochemical results demonstrated that pretreatment with WB600/ZD significantly increased MUC2 protein expression in the colon, whereas WB600 did not have the same effect (Figure 3E). Finally, the immunofluorescence results revealed that ZO-1 expression was significantly lower in the SI group than in the CON group, which was prevented by WB600/ZD pretreatment (Figure 3F). Overall, WB600/ZD ameliorates *S*. Infantis infection by enhancing the function of the gut barrier and mucous membrane.

### Oral administration of WB600/ZD prevents *S*. Infantis-induced oxidative stress

**Figure 4:**
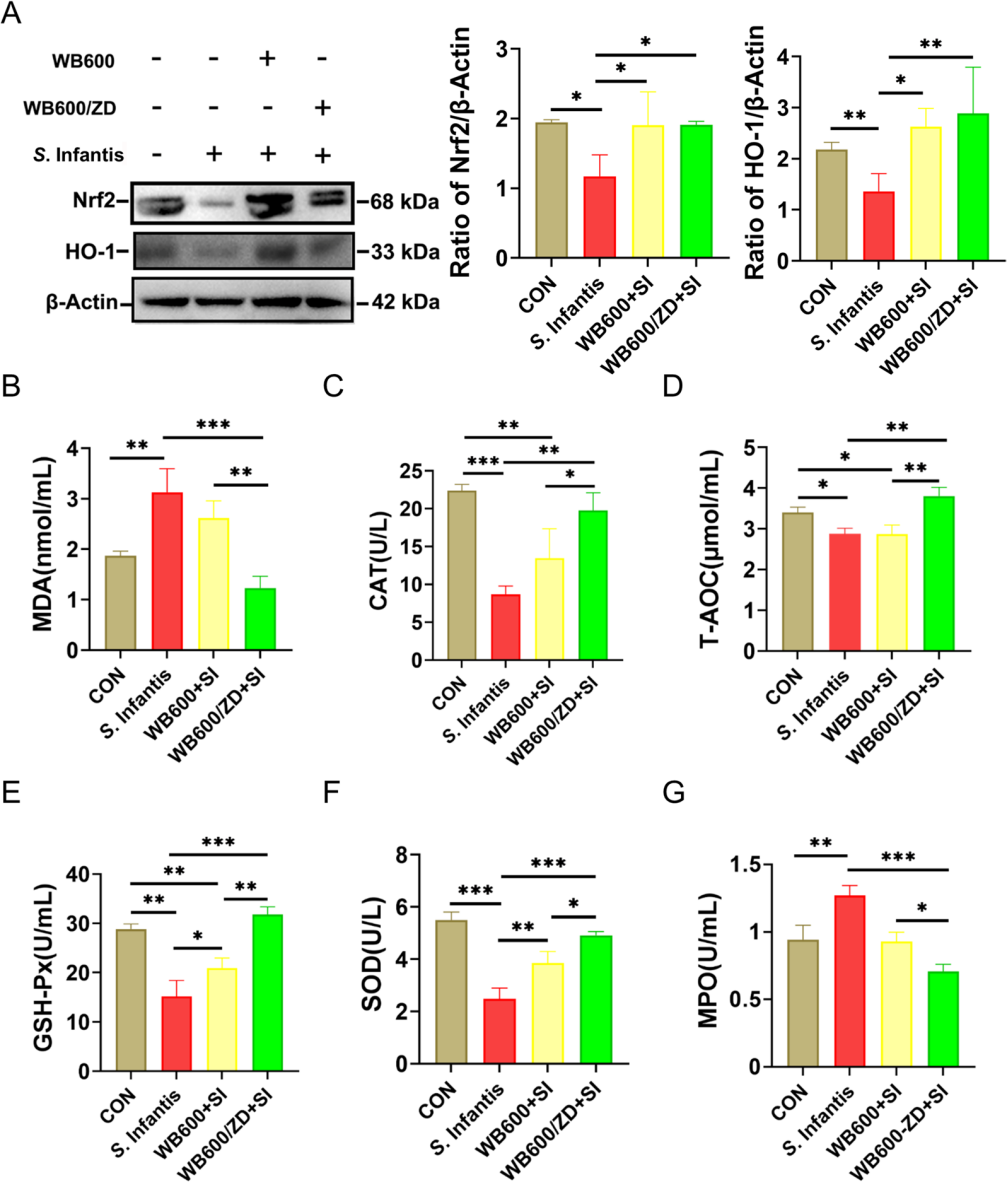
**WB600/ZD prevented oxidative stress caused by** ***S.* Infantis**. **A** Western blot analysis of Nrf2 and HO-1 in the ileum. **B** MDA, **C** CAT, **D** T-AOC, **E** GSH-Px, **F** SOD, and **G** MPO levels in the serum of the mice. All the data are presented as the means ± SD from three independent experiments. *P < 0.05, **P < 0.01, ***P < 0.001.

Compared with those in the CON group, the levels of Nrf2 and HO-1 were significantly restored by WB600/ZD pretreatment (Figure 4A). *S.* Infantis infection increased the concentration of MDA in the serum compared with that in the CON group (Figure 4B). Compared with the SI group, pretreatment with WB600/ZD resulted in a reduction in serum MDA levels. *S.* Infantis infection led to a decrease in T-AOC and the activities of antioxidant enzymes such as CAT, GSH-Px, and SOD (Figures 4C–F). While pretreatment with WB600 increased the serum levels of GSH-Px and SOD, WB600/ZD was more effective at increasing the levels of these antioxidant enzymes. Pretreatment with WB600/ZD prevented the increase in MPO levels induced by *S.* Infantis infection, whereas WB600 pretreatment did not result in the same phenomenon (Figure 4G).

### WB600/ZD pretreatment prevents the inflammatory response in mice with *S*. Infantis infection

**Figure 5:**
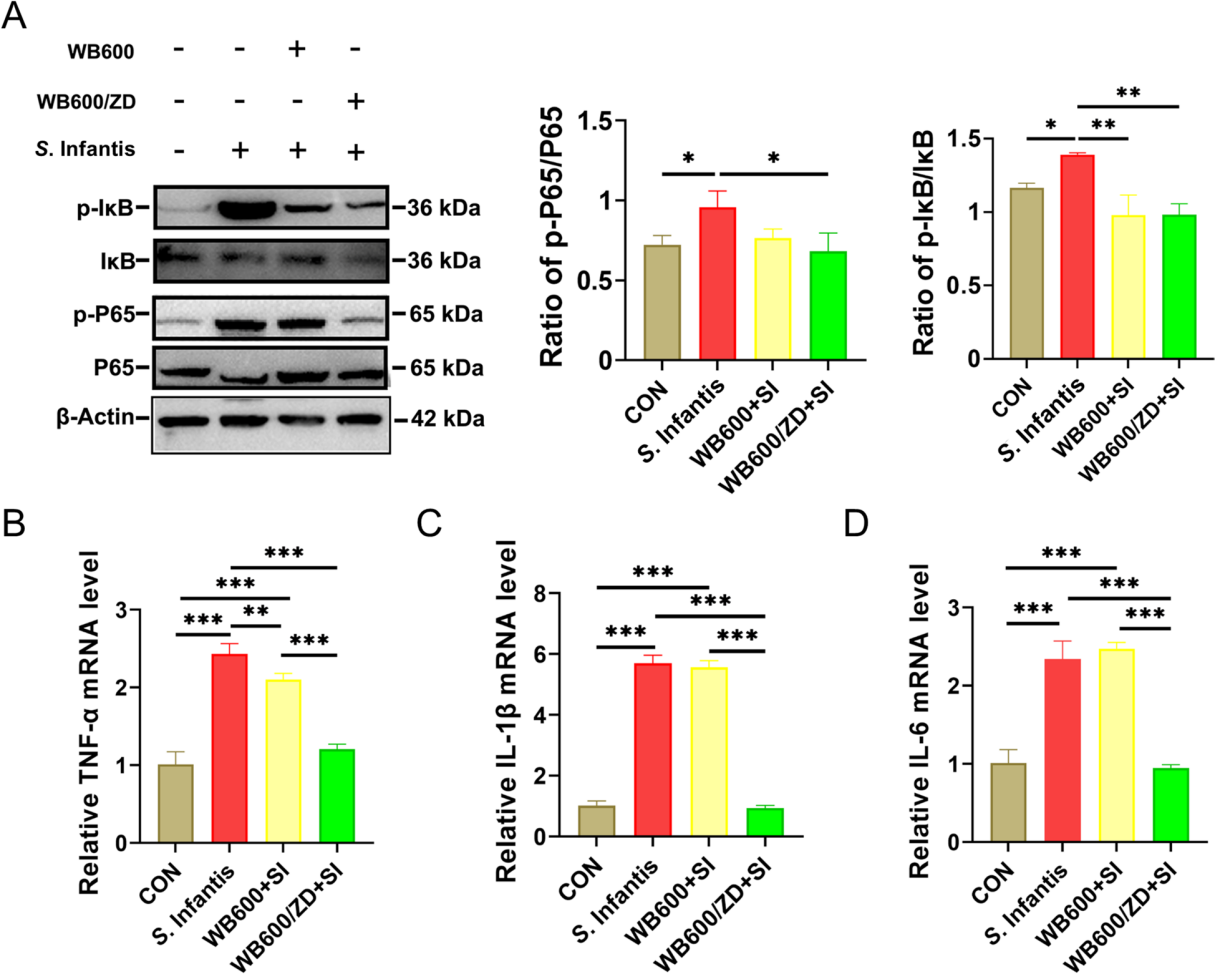
**WB600/ZD inhibited inflammatory responses caused by** ***S.* Infantis infection**. **A** Western blot analysis of IκB, p-IκB, P65, and p-P65 in the ileum. **B**–**D** Relative expression of the proinflammatory genes *TNF-α*, *IL-1β*, and *IL-6* in the ileum. All the data are presented as the means ± SD from three independent experiments. *P < 0.05, **P < 0.01, ***P < 0.001.

We investigated the effects of pretreatment with WB600/ZD on the inflammatory response caused by *S*. Infantis infection and reported that pretreatment with WB600/ZD inhibited the activation of the NF-κB pathway induced by *S*. Infantis, as evidenced by the downregulation of p-IκB and p-P65 expression (Figure 5A). In a subsequent investigation, we examined whether WB600/ZD influenced the expression of inflammatory cytokines in *S.* Infantis-induced small intestinal inflammation. The findings revealed that pretreatment with WB600/ZD significantly suppressed the expression of the proinflammatory cytokine genes *TNF-α*, *IL-1β*, and *IL-6* in the ileum (Figures 5B–D) and jejunum (Additional file 5). While pretreatment with the WB600 also improved these indicators to some extent, it was not as effective as pretreatment with the WB600/ZD. Therefore, WB600/ZD was found to attenuate the small intestinal damage induced by *S.* Infantis infection, possibly through the downregulation of the inflammatory response.

### Oral administration of WB600/ZD improves colonic flora diversity in mice

**Figure 6:**
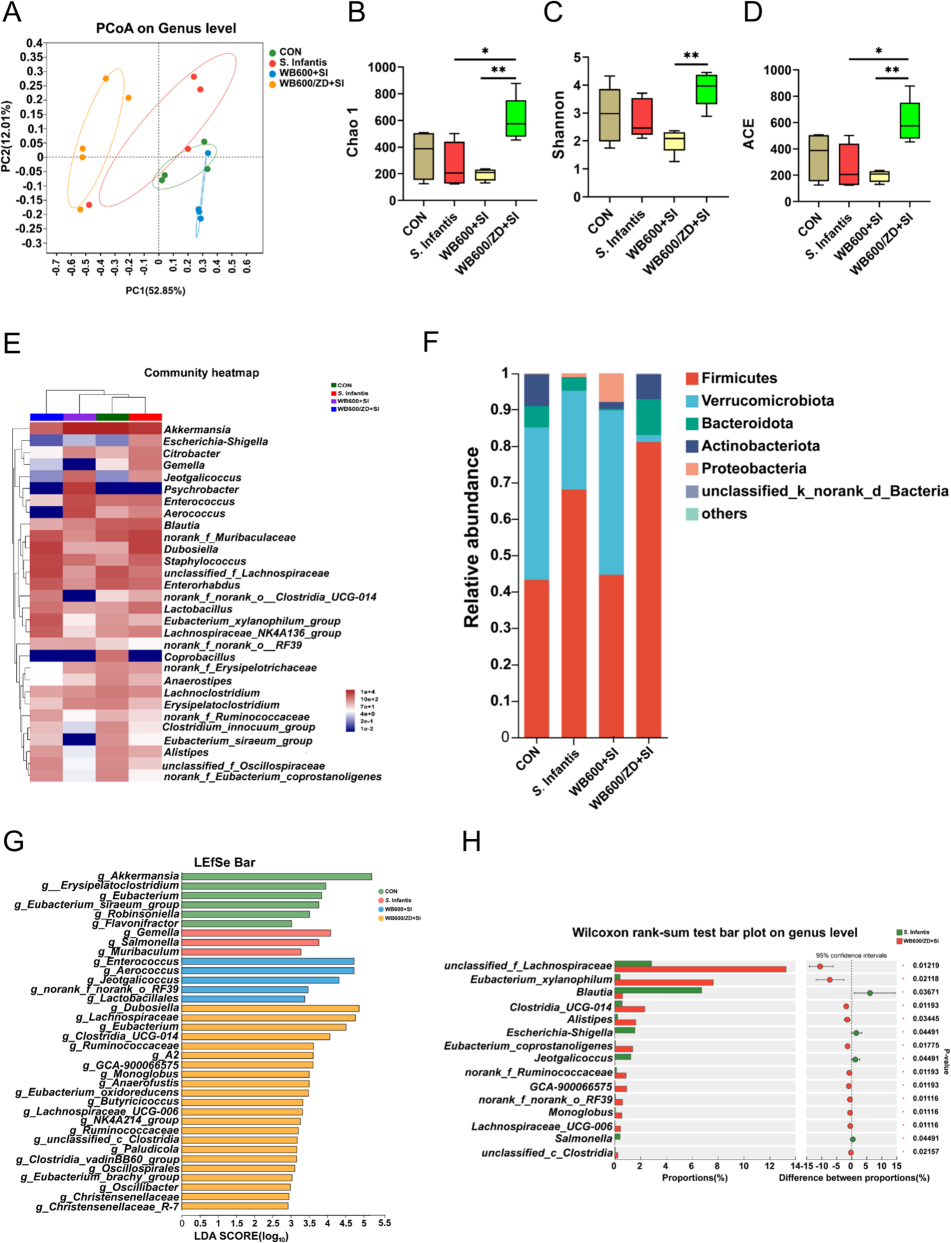
**Pretreatment with WB600/ZD altered the microbial composition of the colonic microbiota in mice challenged with** ***S.*** **Infantis (*n*** **= 5)**. **A** Principal coordinate analysis (PCoA) based on weighted UniFrac analysis. **B**−**D** Alpha diversity indices: Chao1, Shannon, and ACE. **E** Community heatmap. **F** Relative abundance at the genus level. **G** LEfSe at the genus level. **H** Wilcoxon rank-sum test bar plot at the genus level between the SI and the WB600/ZD + SI groups. *P < 0.05, **P < 0.01, ***P < 0.001.

**Figure 7:**
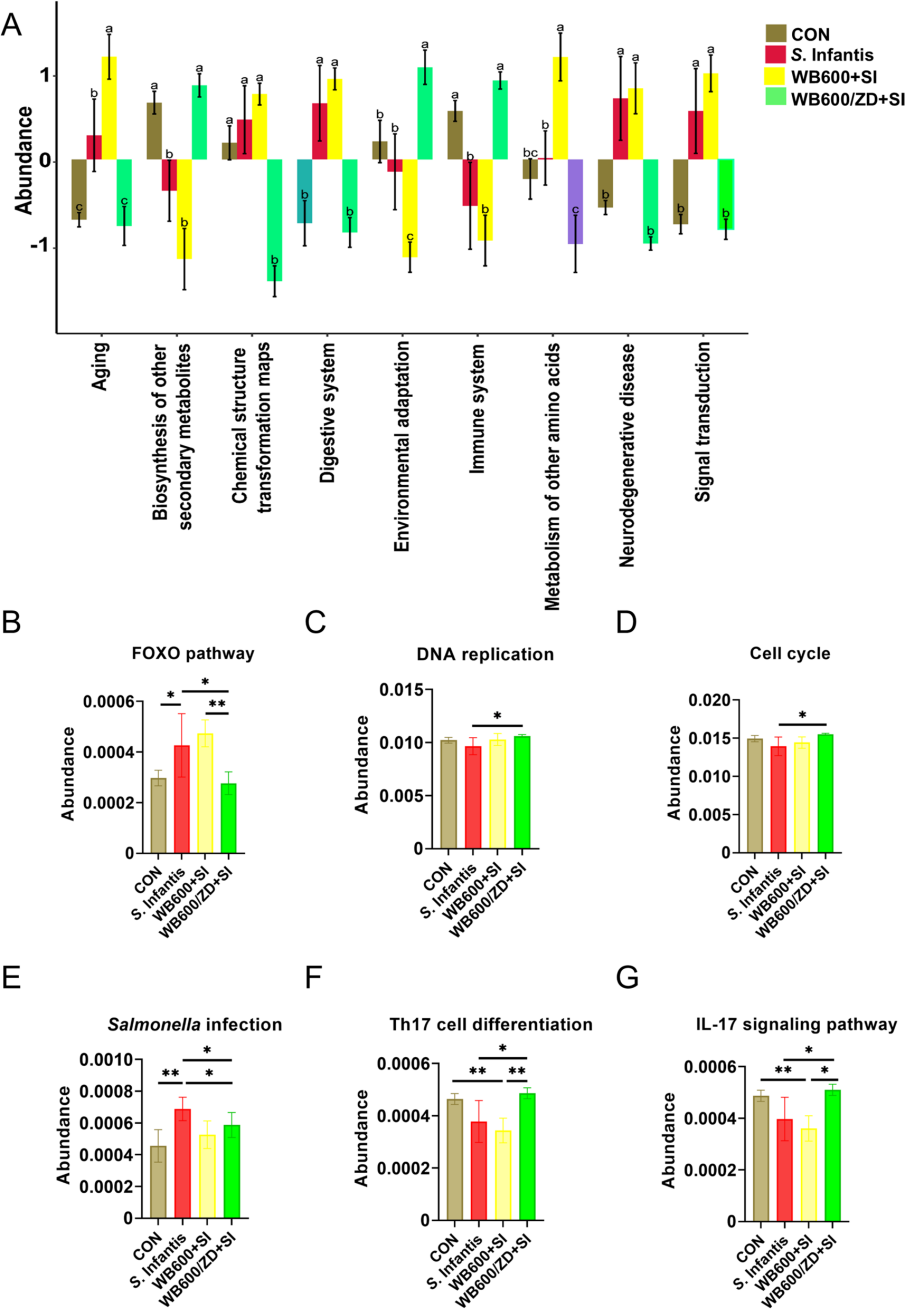
**KEGG pathway of the gut microbiota predicted using phylogenetic investigation of communities by reconstruction of unobserved states (PICRUSt2)**. **A** PICRUSt2 was used to predict the potential mechanism of WB600/ZD for the prevention of *S*. Infantis infection. WB600/ZD alleviated *S*. Infantis infection by **B–D** promoting cell proliferation and **E**–**G** modulating immunity. The data are presented in a bar plot with 95% confidence intervals. *P < 0.05, **P < 0.01, ***P < 0.001.

We further tested the effect of WB600/ZD on the intestinal microbiota. PCoA confirmed that pretreatment with WB600/ZD affected the composition of the intestinal flora in the mice (Figure 6A). Alpha diversity analysis evaluates the richness and diversity of microbial communities in environmental samples via a variety of diversity indices. Parameters such as the Chao 1 estimator, Shannon index, and ACE are crucial for characterizing alpha diversity. Pretreatment with WB600/ZD resulted in a significant increase in all these parameters, suggesting that WB600/ZD supplementation led to greater species abundance within the intestinal flora (Figures 6B–D). We subsequently conducted a detailed analysis of the intestinal flora at the genus and phylum levels. Heatmap analysis was employed to visualize the composition and relative abundance of the intestinal microbial communities, revealing alterations in the microbial community composition induced by both WB600/ZD prefeeding and *S.* Infantis infection (Figure 6E). Community bar plot analysis at the phylum level revealed notable differences among the groups. In the WB600/ZD + SI group, there was an increase in Firmicutes (81%) and Bacteroidota (10%) and a decrease in Verrucomicrobiota (2%) compared with those in the CON group. On the other hand, *S.* Infantis infection resulted in an increase in Verrucomicrobiota (27%) and a decrease in Bacteroidota (3%) (Figure 6F)*.* Compared with the SI treatment, pretreatment with WB600/ZD increased the abundance of probiotics, including *Lachnospiraceae*, *Eubacterium_xylanophilum*, *Clostridia_UCG-014*, and *Alistipes*. It also downregulated the abundance of pathogenic bacteria such as *Escherichia-Shigella* and *Salmonella* (Figures 6G and H), which is consistent with the findings of the fecal loads of *S.* Infantis (Additional file 4), which may be one of the reasons why it affects the disease process. Finally, PICRUSt2 was used to predict the potential mechanism of WB600/ZD for the prevention of *S*. Infantis infection (Figure 7A). The results showed that WB600/ZD was able to affect multiple signal transduction pathways, in particular, by modulating cell proliferation (Figures 7B–D) and immune function (Figures 7E–G), which promoted the clearance of pathogenic bacteria and the repair of intestinal barrier function after intestinal infection.

## Discussion

In this study, we successfully engineered *B. subtilis* WB600/ZD to express and secrete ZD, confirming its protective effects against *S*. Infantis-induced intestinal inflammation. Our mechanistic investigations revealed that WB600/ZD enhanced intestinal barrier function by increasing the expression of tight junction TJPs and mucosal barrier genes and proteins. Furthermore, WB600/ZD increased the antioxidant capacity in mice. Additionally, WB600/ZD attenuated the inflammatory response by inhibiting the activation of the NF-κB pathway. Importantly, the oral delivery of WB600/ZD enriched the population of beneficial bacteria and reduced the number of pathogens. This promoted microbiota balance and alleviated intestinal inflammation.

AMPs play crucial roles in the innate defense of the intestinal mucosa. Upon bacterial invasion, goblet and Paneth cells release mucus and AMPs, which aid in preventing bacteria from adhering to the epithelial surface and inhibit their proliferation [23, 24]. Multiple studies have shown that supplementation with AMPs can reduce inflammation caused by pathogenic bacteria [25, 26]. However, the instability of AMPs during in vivo delivery restricts their effectiveness in treating intestinal bacterial infections [27]. Here, we established a *B. subtilis* secretion expression system for efficient expression of ZD. Signal peptides are required for protein secretion and expression [28, 29]. Our screening process highlights the critical role of choosing the right signal peptide to optimize protein secretion. It also emphasizes the value of understanding signal peptide properties to improve protein expression systems. Through our screening process, we identified *SPeapD* as the most effective signal peptide, thus establishing a foundation for future research on the heterologous expression of target proteins in *B. subtilis*. Subsequent studies can concentrate on the use of *SPeapD* to secrete a variety of proteins of interest and investigate its applications in biotechnology and industry. The screening process underscores the importance of signal peptide selection in optimizing protein secretion and highlights the importance of understanding signal peptide characteristics for enhancing protein expression systems.

*B. subtilis* significantly enhances the intestinal microbiota balance. During its growth, it produces bioactive compounds such as bacteriocins, polymyxins, mycoplasma, and short bacillus peptides. These substances exert pronounced inhibitory effects on pathogenic bacteria and conditional pathogens that can cause infections under certain conditions [30]. In addition, *B. subtilis* rapidly consumes free oxygen in the intestinal tract, causing intestinal hypoxia, which promotes the growth of beneficial anaerobic bacteria and indirectly inhibits the growth of other pathogenic bacteria [31]. In our previous studies, we reported that ZD is intolerant to enzymes in the digestive tract, especially pepsin and trypsin, in the stomach and small intestine; therefore, we attempted to use engineered strains as vectors for the gastrointestinal delivery of ZD. To determine the specific role of ZD, we optimized the structure of ZD. Therefore, *B. subtilis* WB600 was selected as a ZD expression and delivery vector. Although WB600 was found to have a certain effect on *S.* Infantis-induced intestinal inflammation, the effects were much weaker than those of WB600/ZD, which confirms that ZD plays an important role in the prevention of *S.* Infantis infection. To our knowledge, this study represents the first application of ZD-producing *B. subtilis* WB600 to prevent intestinal inflammation in vivo.

Our previous studies revealed that probiotics play an important role in the treatment of *S.* Infantis infection [18–21]. *Lactobacillus fermentum* alleviates the damage to the intestinal morphology induced by *S.* Infantis infection by modulating immune function in chickens [14]. In addition, *L. rhamnosus* and *L. johnsonii* can alleviate the intestinal damage caused by *S.* Infantis in pigs by regulating the intestinal flora and producing beneficial metabolites, such as short-chain fatty acids [15, 22].

IECs are connected by tight junctions, adhesion junctions, and desmosomes, which together form a barrier preventing the entry of bacteria, viruses, and endotoxins [32, 33]. When tight junctions in intestinal IECs are compromised, the intestinal barrier is weakened, increasing susceptibility to microbial invasion of the intestinal mucosa, which significantly contributes to intestinal inflammation [34]. Recent studies have demonstrated that* β*-defensin 118 enhances the expression and cellular localization of ZO-1 to regulate tight junction barrier function [35]. We found that pretreatment with WB600/ZD prevented the *S*. Infantis-induced increase in intestinal barrier dysfunction induced by increasing TJP and mucin gene expression and relieves the clinical symptoms and inflammation caused by *S*. Infantis infection.

Several studies have demonstrated that the ability to increase colonic antioxidant capacity through the activation of Nrf2, which in turn blocks the transduction of the NF-κB pathway, ameliorates DSS-induced colitis [36, 37]. To determine the protective effect of WB600/ZD on the intestinal tract of mice, a mouse enteritis model of *S.* Infantis-infection was established, and the expression of Nrf2 and HO-1 in the ileum of the mice was explored. The results confirmed that the expression levels of Nrf2/HO-1, antioxidant enzymes, T-AOC, and MPO were significantly lower in the SI group than in the CON group. This reduction was prevented by WB600/ZD pretreatment, suggesting that WB600/ZD may protect antioxidant homeostasis by maintaining the Nrf2/HO-1 signalling pathway, thereby alleviating enteritis.

The expression of p-P65 is a sign of NF-κB activation [38]. Activation of the NF-κB signalling pathway enhances the transcription of the *TNF-α*, *IL-6*, and *IL-1β* genes [39]. With the increased production and release of proinflammatory cytokines, the NF-κB signalling pathway is activated again, leading to further amplification of the initial inflammatory signals and exacerbating organismal injury and microcirculation disorders [40]. Previous studies have demonstrated that HO-1 can inhibit the transcription of proinflammatory cytokines by blocking NF-κB activation [41]. The results of the present study demonstrated that *S*. Infantis infection caused oxidative damage and activation of the NF-κB signalling pathway, which was reversed by WB600/ZD pretreatment, suggesting that WB600/ZD may inhibit the NF-κB pathway through activation of Nrf2/HO-1, which alleviates enteritis.

The intestinal flora plays an important role in maintaining intestinal health in both humans and animals [33, 42], and maintaining intestinal flora homeostasis can alleviate intestinal diseases, including ulcerative colitis (UC) [43], inflammatory bowel disease (IBD) [44], and bacterial infection [45]. Our results showed that prefeeding with WB600/ZD and *S.* Infantis infection affected the intestinal microbial composition. *S.* Infantis infection did not affect alpha diversity compared with that in the CON group, but pretreatment with WB600/ZD increased host intestinal flora abundance and diversity and directly suppressed the abundance of *Salmonella*, which mitigated the development of intestinal inflammation. Many AMPs have antioxidant activity that maintains redox balance and reduces reactive oxygen species in the gut, which plays an important role in regulating the intestinal flora [46]. In this study, we found that WB600/ZD activated the Nrf2/HO-1 pathway, alleviated *S*. Infantis-induced oxidative damage, and reduced intestinal inflammatory responses. This effect may be associated with the maintenance of intestinal flora homeostasis. Although WB600 also affects the redox capacity of the organism, its effect is much less than that of WB600/ZD, which suggests that ZD plays a more important role in alleviating *S.* Infantis infection, but, the specific interaction mechanisms between these two pathways need to be further studied.

In conclusion, our findings suggest that enteral delivery of ZD via WB600/ZD may be a valuable approach for reducing intestinal inflammation. It can prevent clinical symptoms and pathological damage caused by *Salmonella* infection in mice and restore intestinal tight junction and mucin expression. In addition, preadministration of WB600/ZD improved systemic antioxidant capacity and reduced the inflammatory response by activating the Nrf2/HO-1 pathway and inhibiting the NF-κB pathway in response to *S.* Infantis. Finally, WB600/ZD pretreatment improved the intestinal flora disorder caused by *Salmonella* infection and reduced the abundance of harmful bacteria. Our study provides a theoretical basis for new therapeutic approaches for bacterial intestinal inflammation.

## Supplementary Information

**Additional file 1. Sequences of oligonucleotide primers used for plasmid construction**. The table shows information on the oligonucleotide primers used for plasmid construction in this study.

**Additional file 2. Sequences of oligonucleotide primers used for quantitative real-time PCR**. The table shows information on the oligonucleotide primers used for quantitative real-time PCR in this study.

**Additional file 3. WB600/ZD secretes ZD**. (A) Positive transformants of WB600/ZD. (B) PCR amplification of five signal peptide genes (*bpr*, *dacB*, *eapD*, *lytE*, *wapA*). (C) Bacteriostatic effect of WB600/ZD fermentation broth supernatant on pathogenic bacteria, *S*. Infantis, *E. coli* ATCC25922, *Klebsiella pneumoniae*, *Staphylococcus aureus* ATCC33591, ATCC25923, ATCC29213, and *E. coli* CAU201919. 1-4 represent ampicillin, WB600/ZD culture medium supernatant, PBS and WB600 culture medium supernatant, respectively.

**Additional file 4. WB600/ZD pretreatment reduced the faecal and organ** ***S***.** Infantis loads**. (A) Fecal *S*. Infantis load at days 1–3 after *S*. Infantis infection. (B) Liver *S*. Infantis load and (C) spleen *S*. Infantis load on day 21. All the data are presented as the means ± SD from three independent experiments. *P < 0.05, **P < 0.01, ***P < 0.001.

**Additional file 5. WB600/ZD promotes the expression of intestinal mucin-related genes and prevents the expression of proinflammatory cytokines in** ***S.* Infantis-induced intestinal inflammation**. The relative mRNA levels of (A) *MUC2*, (B) *TFF3*, and (C) *GAL3ST2* in the jejunum were determined by RT‒qPCR. The relative mRNA levels of (D) *TNF-α*, (E) *IL-1β*, and (F) *IL-6* in the jejunum were determined by RT‒qPCR. All the data are presented as the means ± SD from three independent experiments. *P < 0.05, **P < 0.01, ***P < 0.001.

## Data Availability

The data that support the findings of this study are available from the corresponding author upon reasonable request.

## Abbreviations

AMPs: antimicrobial peptides
ZD: *Zophobas atratus* Defensin
MUC2: mucin 2
TFF3: trefoil factor 3
GAL3ST2: galactose-3-O-sulfotransferase 2
IL: interleukin
TNF-α: tumor necrosis factor-α
Nrf2: nuclear factor erythroid-related factor 2
HO-1: heme Oxygenase-1
H&E: hematoxylin and eosin
TJPs: tight junction proteins
NF-κB: nuclear factor kappa-B
SP: signal peptide
