# Supplementary material for: Engineered Bacillus subtilis WB600/ZD prevents Salmonella Infantis-induced intestinal inflammation and alters the colon microbiota in a mouse model
Source: Vet Res. 2025 Feb 8;56:35. doi: 10.1186/s13567-024-01438-z (PMC11806837; doi:10.1186/s13567-024-01438-z)
Supplement: Supplementary file 1 — Additional file 1. Sequences of oligonucleotide primers used for plasmid construction. The table shows information on the oligonucleotide primers used for plasmid construction in this study. [file 13567_2024_1438_MOESM1_ESM.docx]

**Additional file 1. Sequences of oligonucleotide primers used for** **plasmid construction**

| Primer name | Primer sequence (5'-3') | Product length (bp) |
| --- | --- | --- |
| SP-bpr-F | aaagtgaaatcagggggatccATGAGGAAAAAAACGAAAAACAGAC | 130 |
| SP-bpr-R | agtggtggtgatggtggtgTGCCCCGGCTGCTCCCGG |  |
| bpr-zd | aCACCACCATCACCACCACTTC | 367 |
| SP-dacB-F | aaagtgaaatcagggggatccATGCGCATTTTCAAAAAAGCA | 108 |
| SP-dacB-R | gtggtgAGCATGTGCTGTATTCACATTTACG |  |
| dacB-zd-F | atacagcacatgctCACCACCATCACCACCACTT | 339 |
| SP-eapD-F | aaagtgaaatcagggggatccATGAAAAAGCTTTTGACTGTCATGA | 104 |
| SP-eapD-R | tgatggtggtgACTCTGTGCCGGCAAGAGC |  |
| eapD-zd | gcacagagtCACCACCATCACCACCACTTC | 342 |
| SP-lytE-F | aaagtgaaatcagggggatccATGAAAAAGCAAATCATTACAGCTACG | 108 |
| SP-lytE-R | tgatggtggtgTGCAGATGCCGCTCCTGC |  |
| lytE-zd | gcatctgcaCACCACCATCACCACCACTTC | 342 |
| SP-wapA-F | aaagtgaaatcagggggatccATGAAAAAAAGAAAGAGGCGAAAC |  |
| SP-wapA-R | gtgGGCTGGCACTAATGAAATCATTAA | 108 |
| wapA-zd | tttcattagtgccagccCACCACCATCACCACCACTTC | 342 |
| ZD-F | aaaacatcagccgtaggatccCACCACCATCACCACCACTTC | 150 |
| ZD-R | gacgtcgactctagaggatccTTAACGGCAAACGCATACAGAT |  |
| PMA5-F | GCAGAGCACACACTTTATGAAT | 614 |
| PMA5-R | TGGCAAGTGTAGCGGTCAC |  |

The table shows information of oligonucleotide primers used for plasmid construction in this study.
