# Supplementary material for: Engineered Bacillus subtilis WB600/ZD prevents Salmonella Infantis-induced intestinal inflammation and alters the colon microbiota in a mouse model
Source: Vet Res. 2025 Feb 8;56:35. doi: 10.1186/s13567-024-01438-z (PMC11806837; doi:10.1186/s13567-024-01438-z)
Supplement: Supplementary file 2 — Additional file 2. Sequences of oligonucleotide primers used for quantitative real-time PCR. The table shows information on the oligonucleotide primers used for quantitative real-time PCR in this study. [file 13567_2024_1438_MOESM2_ESM.docx]

**Additional file 2. Sequences of oligonucleotide primers used for quantitative real-time PCR.**

| Gene | Direction | Primer sequence (5'-3') |
| --- | --- | --- |
| *GAPDH* | F | TGACCTCAACTACATGGTCTACA |
|  | R | CTTCCCATTCTCGGCCTTG |
| *IL1β* | F | TGCCACCTTTTGACAGTGATG |
|  | R | AAGGTCCACGGGAAAGACAC |
| *TNF-α* | F | ACGGCATGGATCTCAAAGAC |
|  | R | AGATAGCAAATCGGCTGACG |
| *IL-6* | F | CAGAATTGCCATTGCACAAC |
|  | R | CAGAATTGCCATTGCACAAC |
| *MUC2* | F | ATGCCCACCTCCTCAAAGAC |
|  | R | GTAGTTTCCGTTGGAACAGTGAA |
| *TFF3* | F | TTGCTGGGTCCTCTGGGATAG |
|  | R | TACACTGCTCCGATGTGACAG |
| *GAL3ST2* | F | CTGTGTTCCTCCTGGTTGGTTTCC |
|  | R | CTGGCTGTCTTGTGCGTCTTGAG |

The table shows information of oligonucleotide primers used for quantitative real-time PCR in this study.
