# Supplementary figures and images for: Engineered Bacillus subtilis WB600/ZD prevents Salmonella Infantis-induced intestinal inflammation and alters the colon microbiota in a mouse model
Source: Vet Res. 2025 Feb 8;56:35. doi: 10.1186/s13567-024-01438-z (PMC11806837; doi:10.1186/s13567-024-01438-z)

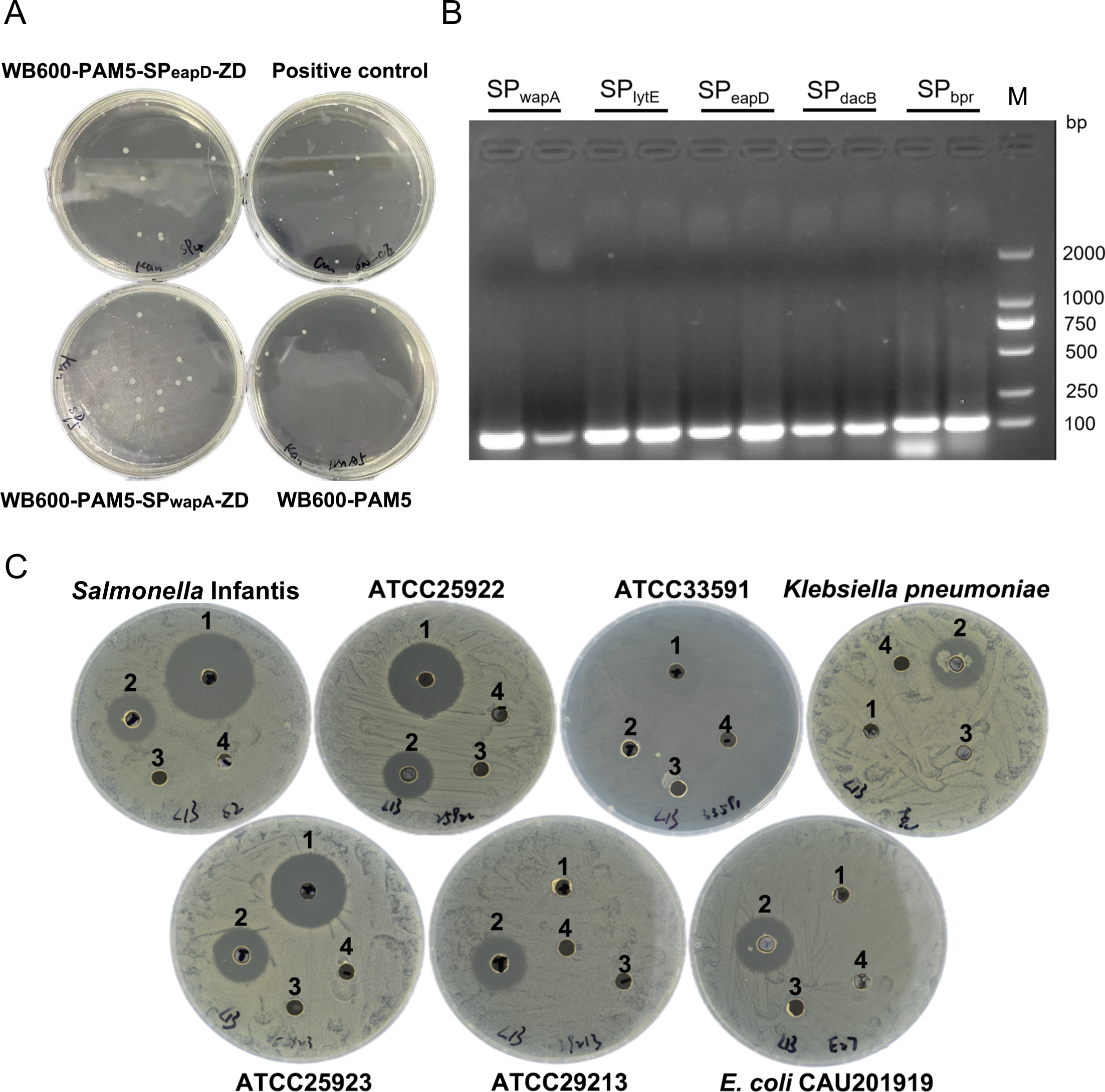

Supplement: Supplementary file 3 — Additional file 3. WB600/ZD secretes ZD. (A) Positive transformants of WB600/ZD. (B) PCR amplification of five signal peptide genes (bpr, dacB, eapD, lytE, wapA). (C) Bacteriostatic effect of WB600/ZD fermentation broth supernatant on pathogenic bacteria, S. Infantis, E. coli ATCC25922, Klebsiella pneumoniae, Staphylococcus aureus ATCC33591, ATCC25923, ATCC29213, and E. coli CAU201919. 1-4 represent ampicillin, WB600/ZD culture medium supernatant, PBS and WB600 culture medium supernatant, respectively. [file 13567_2024_1438_MOESM3_ESM.tif]

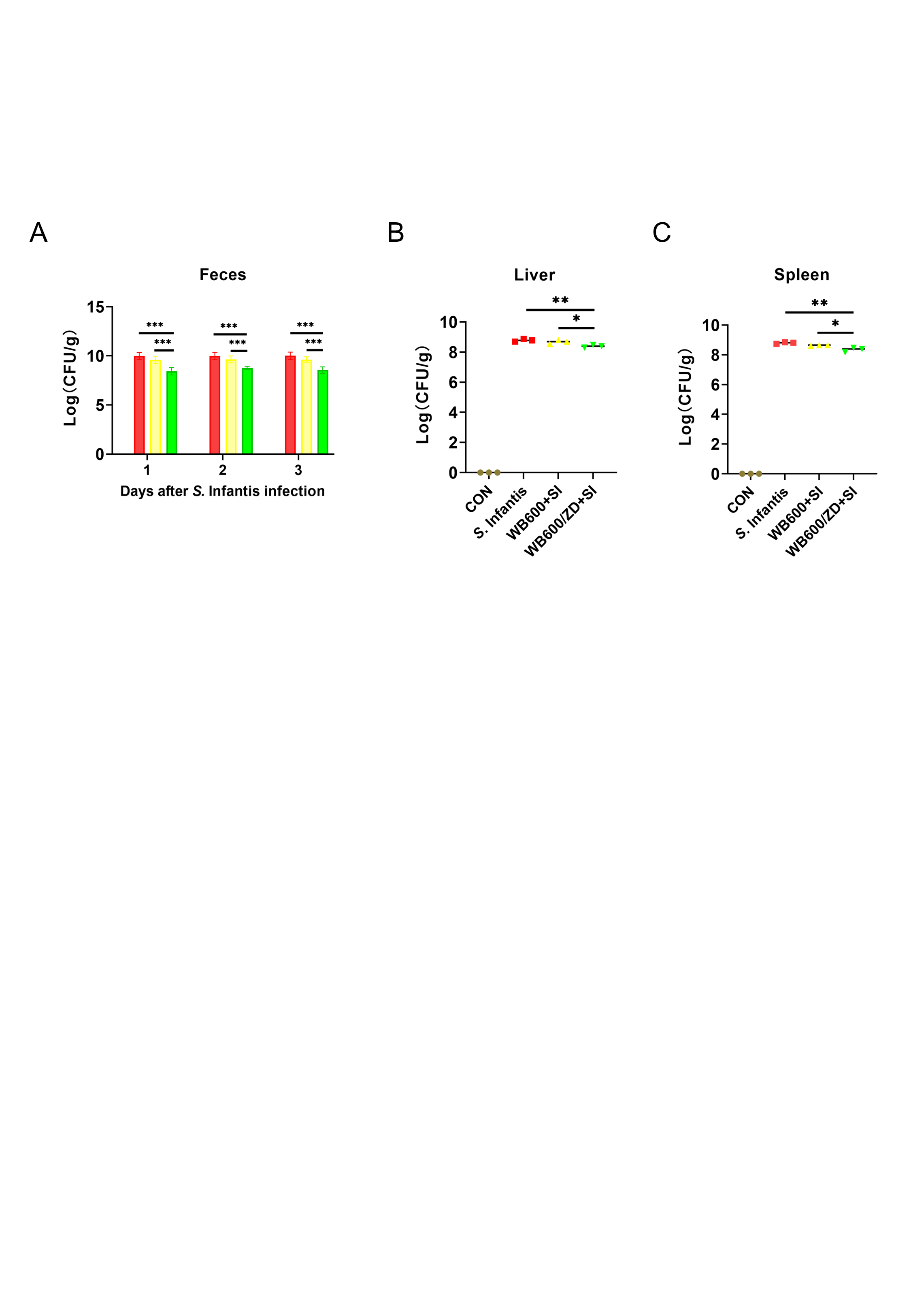

Supplement: Supplementary file 4 — Additional file 4. WB600/ZD pretreatment reduced the faecal and organ S. Infantis loads. (A) Fecal S. Infantis load at days 1–3 after S. Infantis infection. (B) Liver S. Infantis load and (C) spleen S. Infantis load on day 21. All the data are presented as the means ± SD from three independent experiments. *P < 0.05, **P < 0.01, ***P < 0.001. [file 13567_2024_1438_MOESM4_ESM.tif]

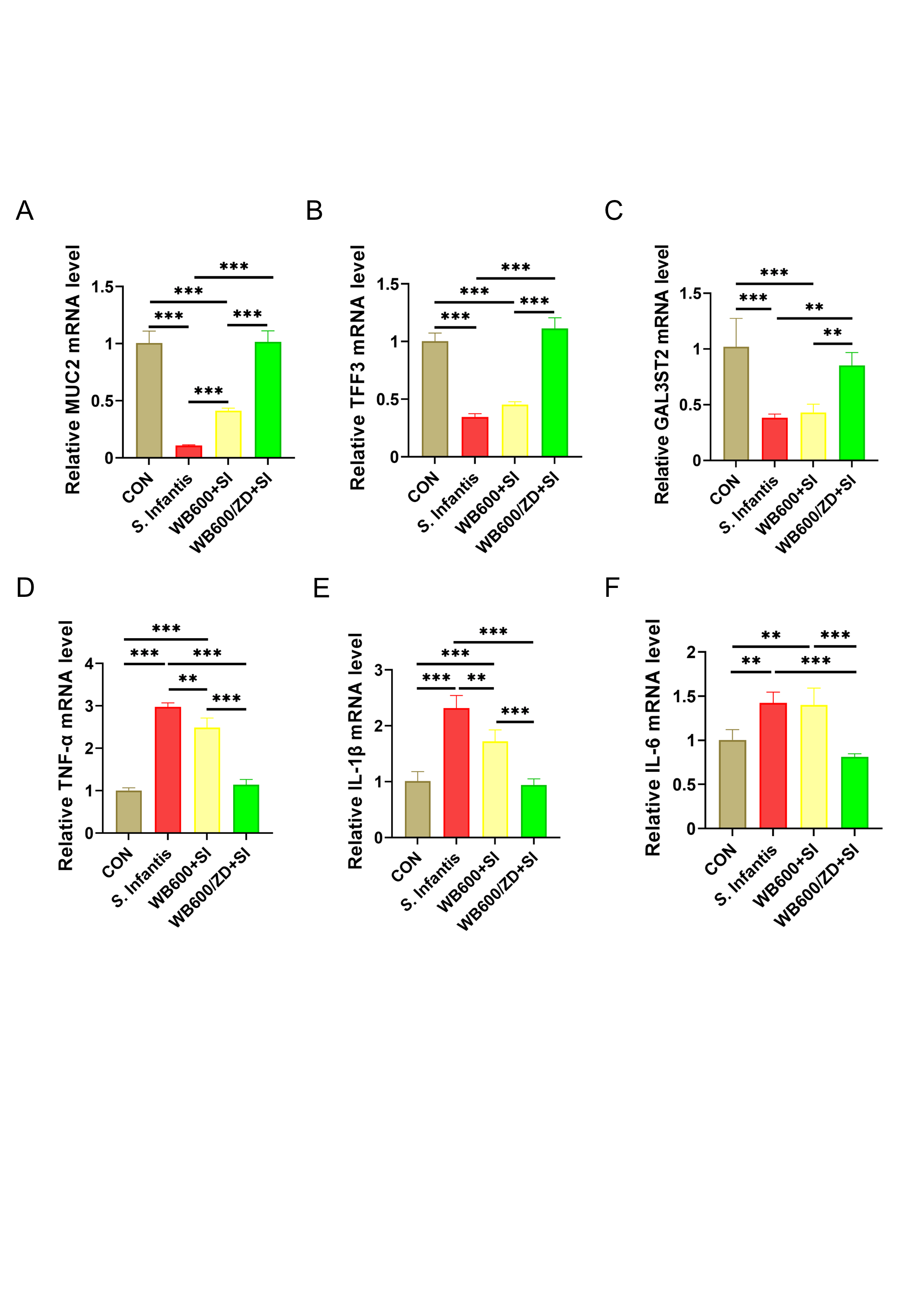

Supplement: Supplementary file 5 — Additional file 5. WB600/ZD promotes the expression of intestinal mucin-related genes and prevents the expression of proinflammatory cytokines in S. Infantis-induced intestinal inflammation. The relative mRNA levels of (A) MUC2, (B) TFF3, and (C) GAL3ST2 in the jejunum were determined by RT‒qPCR. The relative mRNA levels of (D) TNF-α, (E) IL-1β, and (F) IL-6 in the jejunum were determined by RT‒qPCR. All the data are presented as the means ± SD from three independent experiments. *P < 0.05, **P < 0.01, ***P < 0.001. [file 13567_2024_1438_MOESM5_ESM.tif]
